# Acute effect of endurance exercise on human milk insulin concentrations: a randomised cross-over study

**DOI:** 10.3389/fnut.2024.1507156

**Published:** 2025-01-27

**Authors:** Rebecca Lyng Holm, Mads Holmen, Md Abu Jafar Sujan, Guro F. Giskeødegård, Trine Moholdt

**Affiliations:** ^1^Department of Circulation and Medical Imaging, Norwegian University of Science and Technology, Trondheim, Norway; ^2^Department of Obstetrics and Gynaecology, St. Olavs Hospital, Trondheim, Norway; ^3^Department of Public Health and Nursing, Norwegian University of Science and Technology, Trondheim, Norway

**Keywords:** obesity, lactation, metabolism, high-intensity interval training, infant, nutrition, running

## Abstract

**Introduction:**

Insulin is present in human milk and its concentration correlates with maternal circulating levels. Studies on the association between human milk insulin concentrations and infant weight or growth show conflicting results, but some studies indicate that higher insulin concentrations in the milk can promote infant weight gain. Circulating levels of insulin decrease acutely after exercise, but no prior study has investigated the acute effect of exercise on human milk insulin concentrations. Our aim was to determine the acute effects of two endurance exercise protocols on human milk insulin concentration in exclusively breastfeeding individuals.

**Methods:**

In a randomised cross-over trial, 20 exclusively breastfeeding participants who were 6–12 weeks postpartum completed three conditions on separate days: (1) moderate-intensity continuous training (MICT), (2) high-intensity interval training (HIIT), and (3) no activity (REST). Milk was collected before exercise/rest (at 07:00 h), immediately after exercise/rest (11:00 h), 1 h after exercise/rest (12:00 h), and 4 h after exercise/rest (15:00 h). We determined insulin concentrations in the milk using enzyme-linked immunosorbent assay and compared insulin concentrations after MICT and HIIT with REST using a linear mixed model with time-points and the interaction between time and condition as fixed factors.

**Results:**

We detected insulin in all 240 samples, with an average concentration of 12.3 (SD 8.8) μIU/mL (range 3.2–57.2 μIU/mL). There was no statistically significant effect of exercise on insulin concentration, but a tendency of reduced concentrations 4 h after HIIT (*p* = 0.093). There was an overall effect of time at 11:00 h and 15:00 h. In the fasted sample obtained at 07:00 h, the concentration was 9.9 (SD 7.2) μIU/mL, whereas the concentration was 12.7 (SD 9.0) μIU/mL at 11:00 h (*p* = 0.009), and 15.0 (SD 11.7) μIU/mL at 15:00 h (*p* < 0.001).

**Conclusion:**

One session of endurance exercise, either at moderate- or high intensity, had no statistically significant effect on human milk insulin concentration. Future research should determine the effect of regular exercise on insulin in human milk and potential impact for infant health outcomes.

**Clinical trial registration:**

ClinicalTrials.gov, identifier NCT05042414.

## 1 Introduction

The prevalence of overweight and obesity among children and adolescents aged 5–19 has more than doubled over the past three decades, from 8% in 1990 to 20% in 2022 (1). Up to 21% of childhood overweight/obesity is attributable to maternal obesity (2). This mother-to-child transmission of obesity is not purely genetic but involves complex interactions between genes and an “obesogenic” environment, leading to epigenetic modifications (3). During the first 1,000 days of life, including the time in the womb and up to the age of 2 years, genes are especially susceptible to epigenetic modifications that regulate gene expression and thus phenotype (3). Consequently, nutrition during these 1,000 days is crucial for the future susceptibility to obesity. The World Health Organization recommends that infants are exclusively breastfed for the first 6 months of life to prevent overweight/obesity (1). However, concentration of nutrients and bio-active molecules in the milk may vary between mothers according to their body mass index (BMI), with a potential impact of differences in composition on the mother-to-child transmission of obesity (4). We recently proposed that exercise may improve human milk composition and thereby reduce the transmission of obesity from mother to child (5), and showed increased concentrations of adiponectin in human milk acutely after high-intensity exercise (6).

The concentration of insulin in human milk is associated with maternal circulating levels of this hormone (7). Plasma insulin concentrations decrease progressively during exercise (8). However, no prior study has, to our knowledge, investigated the impact of exercise on human milk concentrations of insulin. There is robust evidence supporting a positive correlation between maternal BMI and human milk concentrations of insulin (9). The concentration of insulin in human milk may influence infant growth. In agreement with insulin’s anabolic function, promoting cellular intake of glucose in muscle and adipose tissue, its concentrations in human milk were shown to correlate with infant weight or fat mass index (10, 11). In contrast, others have found no associations between human milk insulin levels and infant body composition (12), yet others a negative correlation (13, 14).

Previous research has indicated that the nutritive composition of human milk is not impacted by a single session of exercise or exercise training (15, 16). However, there has been little investigation of the effect of exercise on other bioactive factors in human milk, such as hormones that regulate metabolism and growth. We aimed to determine the acute effect of endurance training with moderate- and high intensity on human milk insulin concentrations among exclusively breastfeeding individuals. Our hypothesis was that insulin concentrations would decrease following exercise and that there would be a greater effect of high-intensity exercise than of moderate-intensity training.

## 2 Methods

### 2.1 Experimental design, participants, and experimental procedures

**Figure 1:**
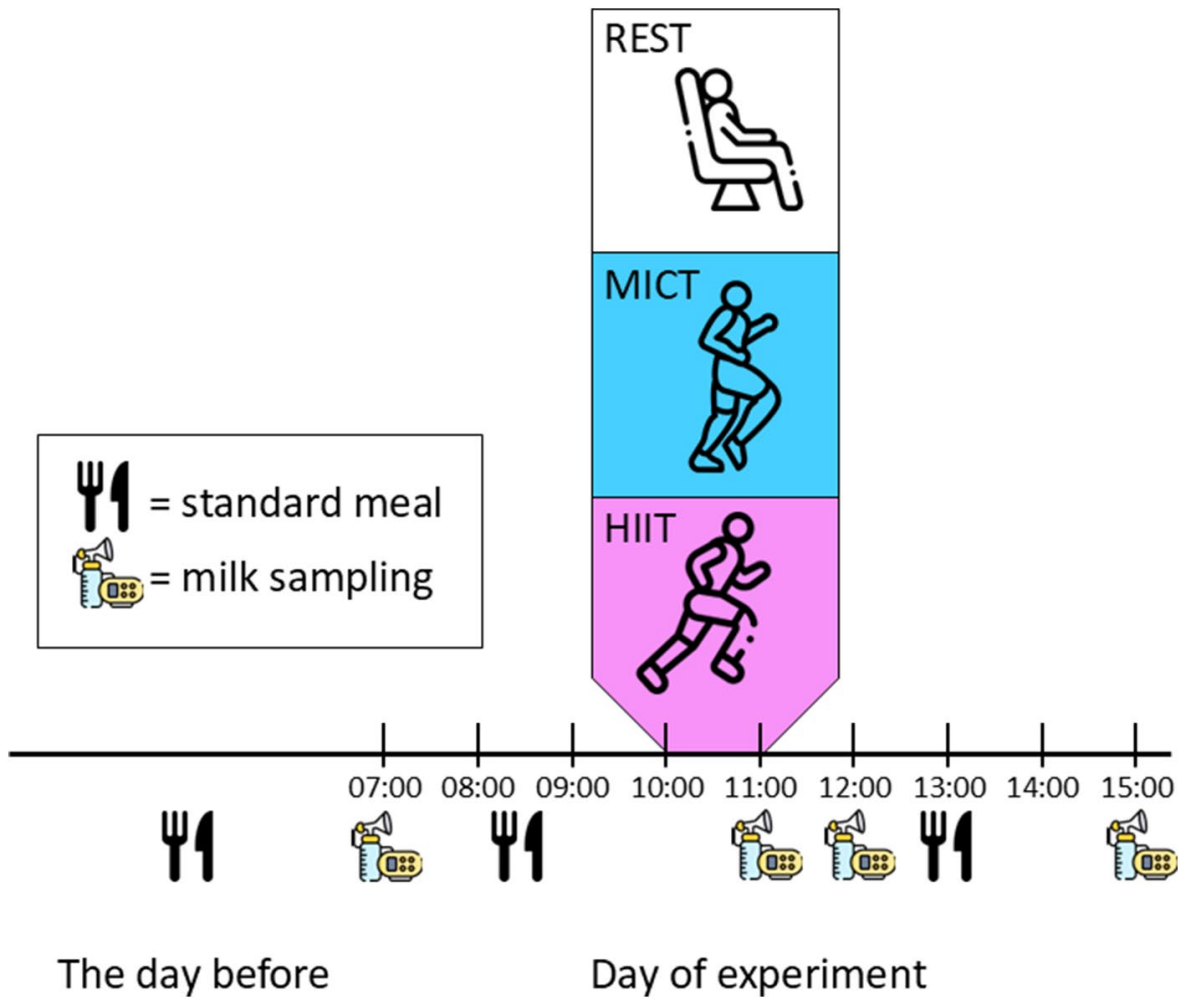
Study design. Participants completed three conditions in random order. REST, no activity; MICT, moderate-intensity continuous training; HIIT, high-intensity interval training. Human milk samples were collected at 07:00 h, 11:00 h, 12:00 h, and 15:00 h at all conditions. The conditions were separated by >48 h, and the participants consumed standardised meals on test days and the night before.

This randomized cross-over study was conducted at the Norwegian University of Science and Technology, in Trondheim, Norway. The study was approved by the Regional Committee for Medical and Health Research Ethics (REK-263493) and pre-registered in clinicaltrials.gov (NCT05042414, 13/09/2021). Results concerning the effect of exercise on human milk adiponectin concentrations have been published previously (6). Inclusion criteria were females aged ≥18 years, exclusively breastfeeding a singleton, term infant aged 6–12 weeks, living in the Trondheim area, and being able to walk or run on a treadmill for >50 min. Exclusion criteria were known cardiovascular disease or diabetes mellitus type 1 or 2. Gestational diabetes was not an exclusion criterion. All participants signed an informed, written consent prior to assessments. After baseline assessments, the participants underwent three conditions in random order: REST (sitting), moderate-intensity continuous training (MICT), and high-intensity interval training (HIIT) (Figure 1). There was a washout period between conditions of ≥48 h. A computer random number generator developed at the Faculty of Medicine and Health Science at NTNU was used to randomize the sequence of the conditions for each participant on the first test day. The last author performed the randomization and received the allocation order on screen and by e-mail. The participants did not get information about the sequence of conditions and were only informed about which condition they were undertaking on the test days. Neither participants nor investigators were blinded due to the nature of the intervention (exercise). All methods were performed in accordance with the relevant guidelines and regulations.

On a separate day before the three conditions, we collected data on background characteristics, physical activity levels, body composition, and maximum oxygen uptake (VO_2_max). These assessments were undertaken >48 h before the first condition. We used a bioimpedance scale (InBody720 BioSpace Co., Republic of Korea) to estimate body composition. We measured VO_2_max during a maximal effort exercise test on a treadmill using direct analysis of expired gases (MetaLyzer II, Metasoft, CORTEX Biophysik, Germany). The participants wore heart rate monitors (Polar, Finland) during the exercise testing, and we used the highest recorded heart rate during the test as estimates of individual heart rate maximum (17). The participants completed questionnaires on background characteristics and the International Physical Activity Questionnaire (18). They were requested to avoid exercise >48 h prior to laboratory assessments and experimental procedures. The participants recorded their dietary intake from the evening before the first test day and on the first test day. This recording included the type of foods consumed, amount, and timing of intake. We asked them to repeat the same dietary intake on the later test days, including the type of foods consumed, the amount, and the time of day (Figure 1).

The participants consumed breakfast at 08:30 h and lunch at 13:00 h on all test days, and all three experimental conditions took place at 10:00 h. Immediately after the human milk collection at 11:00 h, the participants consumed a standardised snack. They chose between a banana or a crispbread with cheese and the snack was the same for all conditions for each participant. During the REST condition, the participants rested for 45 min seated in a comfortable chair at the laboratory. MICT was completed as 48 min of walking or jogging at an intensity corresponding to 70% of heart rate maximum, whereas the HIIT protocol was 4 × 4 min HIIT at 90–95% of heart rate maximum, as previously described (6). We used the recorded heart rate with 5-min intervals during MICT and the average heart rate in the last 2 min of every work-bout during HIIT to estimate the actual exercise intensity as a measure of compliance with the exercise protocols.

### 2.2 Human milk sampling and insulin analysis

The participants were supplied with electronic breast pumps (Medela Swing Flex, Medela AG, Switzerland). Human milk was sampled at four time-points on the experimental test days: at 07:00 (before breakfast), 11:00 (immediately after exercise/rest), 12:00 (1 h after exercise/rest), and 15:00 (4 h after exercise/rest) (Figure 1). Only the 07:00 h sample was collected in the fasted state. We asked the participants to provide us with ≥25 mL at each time-point, from the same breast. The participants stored the first (07:00) and last (15:00) samples in their home freezer and transported these on ice to the laboratory. The samples from the remaining time-points were obtained in the laboratory and immediately stored at −80° until analysis. The participants could breastfeed their infant at any time during the test days and we did not control for this in the analysis.

The milk was thawed at room temperature before centrifugation at 10,000 × g for 60 min. We carefully removed the fat layer on the top using tweezers and used the skimmed milk for analysis. We used enzyme-linked immunosorbent assay (ELISA) for quantitative measurement of insulin (IBL International GmBH, Germany, product number RE53171), using a Dynex DS2 automation system programmed with DS-Matrix software (Montebello Diagnostics AS, Norway). The intra-assay variability for the insulin kit is <3% and inter-assay variability is 6%. All samples from each participant were measured in duplicate wells using the same microtiter plate and kit. We reduced the time for the final incubation step to 12 min (instead of 15 min in the manufacturer’s instructions), based on pilot testing of the kits, otherwise, we followed the manufacturer’s instructions. The range of the ELISA assay was 1.76–100 μIU/mL and all measurements were obtained in the linear range of the assay, with a coefficient of determination for the standard curve of 1.

### 2.3 Statistical analysis

We did not do a formal sample size calculation due to the exploratory nature of the research question but aimed to include 20 participants. The advantage of cross-over studies is that it allows comparison at the individual level rather than the group level and fewer participants are required in a cross-over design compared with a parallel group design. To determine the effect of HIIT and MICT on human milk adiponectin concentrations, we used a linear mixed model with time and the interaction between time and condition as fixed effects and participant ID as random effects. The first time-point (07:00 h) and REST were used as reference categories for time and condition, respectively. Data were log-transformed to achieve normally distributed residuals. We consider *p*-values <0.05 as statistically significant. We used IBM SPSS 29.0.1.0 for the statistical analysis. Conditional effect estimates were calculated by exponentiating the coefficients from the linear mixed model analysis, which gives multiplicative factors and represents estimated effects in individuals with equal random intercepts.

## 3 Results

### 3.1 Participants and compliance with exercise protocols and human milk sampling

**Figure 2:**
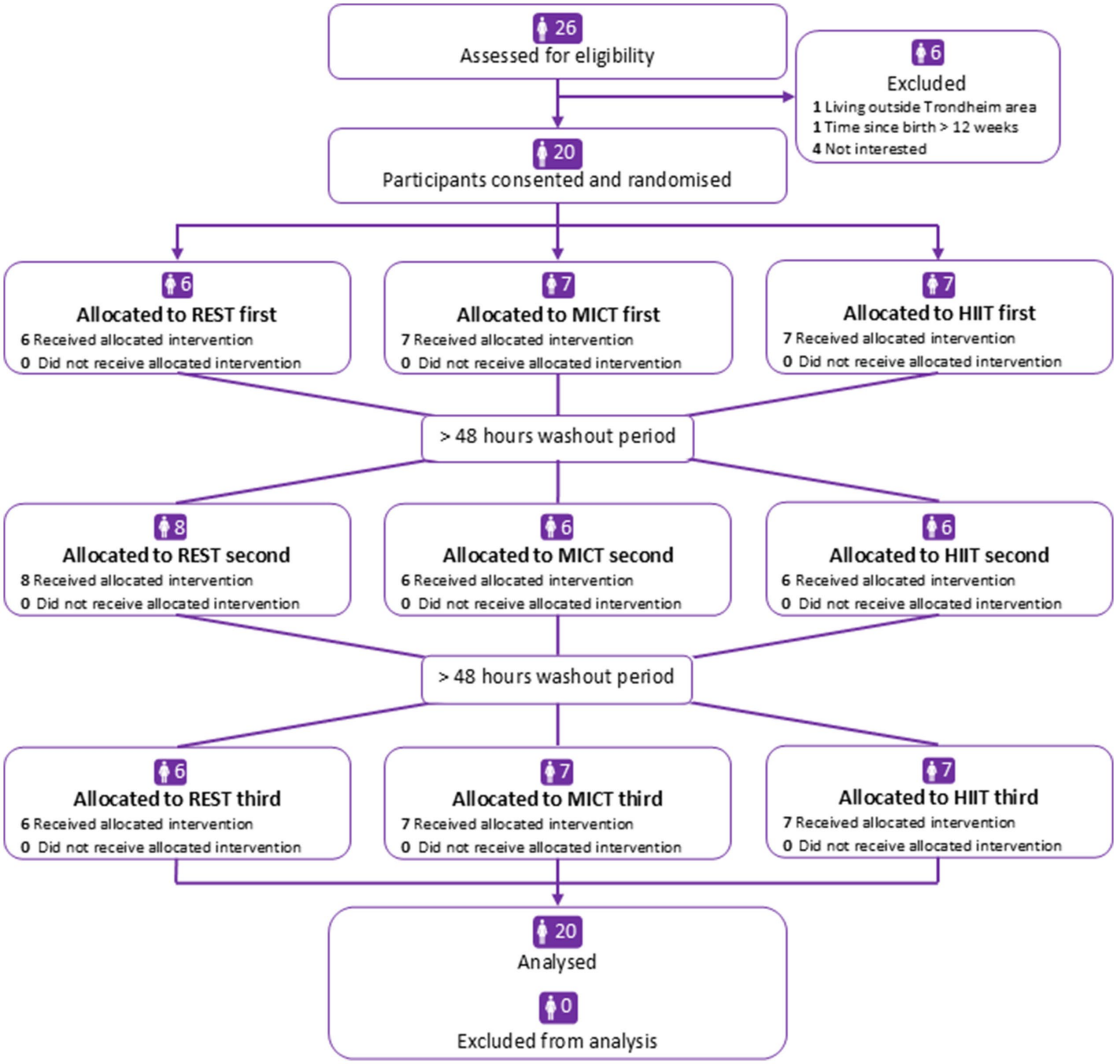
Participant flowchart. REST, no activity; MICT, moderate-intensity continuous training; HIIT, high-intensity interval training.

Recruitment began in August 2021 and concluded in May 2022. We ended the recruitment when we had reached the pre-specified number of participants that we aimed to include. We included 20 participants, who completed all three conditions (Figure 2). Table 1 shows their baseline characteristics. No adverse events were reported. Insulin was detected in all milk samples, with an average concentration of all 240 samples of 12.3 (SD 8.8) μIU/mL (range 3.2–57.2 μIU/mL). Within the 12 samples obtained from each participant, the average SD was 5.2 μIU/mL (Supplementary Table S1). Table 2 shows the observed data for insulin concentrations at each time-point in the three conditions.

**Table 1:** Baseline characteristics of participants, by which condition they completed first.

	REST (*n* = 6)	MICT (*n* = 7)	HIIT (*n* = 7)
Age, years	31 (3)	30 (2)	32 (3)
Body mass, kg	77.8 (9.6)	66.0 (6.2)	73.6 (8.1)
Body mass index, kg/m^2^	28.3 (3.8)	24.3 (3.0)	26.2 (3.3)
Fat mass, kg	28.6 (9.3)	18.1 (7.3)	24.2 (5.6)
Peak oxygen uptake, mL kg^−1^ min^−1^	34.7 (5.0)	44.0 (7.9)	38.9 (7.8)
Time since delivery, weeks	9.1 (2.2)	8.4 (1.9)	9.3 (1.7)
Infant birth weight, g	3,963 (188)	3,338 (276)	3,700 (493)

Numbers are mean values with standard deviations. REST, no exercise; MICT, moderate-intensity continuous training; HIIT, high-intensity interval training.

**Table 2:** Human milk insulin concentrations at different time-points in the three conditions for 20 participants.

	Days postpartum	07:00 h	11:00 h	12:00 h	15:00 h
REST	69 (13)	8.8 (4.9) μIU/mL	11.9 (6.8) μIU/mL	10.8 (5.4) μIU/mL	15.8 (8.3) μIU/mL
MICT	70 (16)	9.8 (8.4) μIU/mL	12.4 (8.4) μIU/mL	10.9 (7.8) μIU/mL	15.6 (8.5) μIU/mL
HIIT	70 (14)	11.0 (11.5) μIU/mL	13.9 (11.4) μIU/mL	12.8 (11.5) μIU/mL	13.7 (9.5) μIU/mL

Data are observed means with standard deviation (SD). REST, no exercise; MICT, moderate-intensity continuous training; HIIT, high-intensity interval training.

The washout period between conditions was on average 7.3 (SD 2.0) days between the first and second condition and 6.9 (SD 1.4) days between the second and third conditions. For some of the samples, there was a small deviation (average 2.5 min, SD 10.1 min) from the prescribed time-points for sampling. In the MICT condition, the participants exercised at an intensity of 70% (SD 1) of heart rate maximum, whereas the average intensity during the last 2 min of HIIT was 96% (SD 2) of heart rate maximum.

### 3.2 Effect of exercise on insulin concentrations

**Figure 3:**
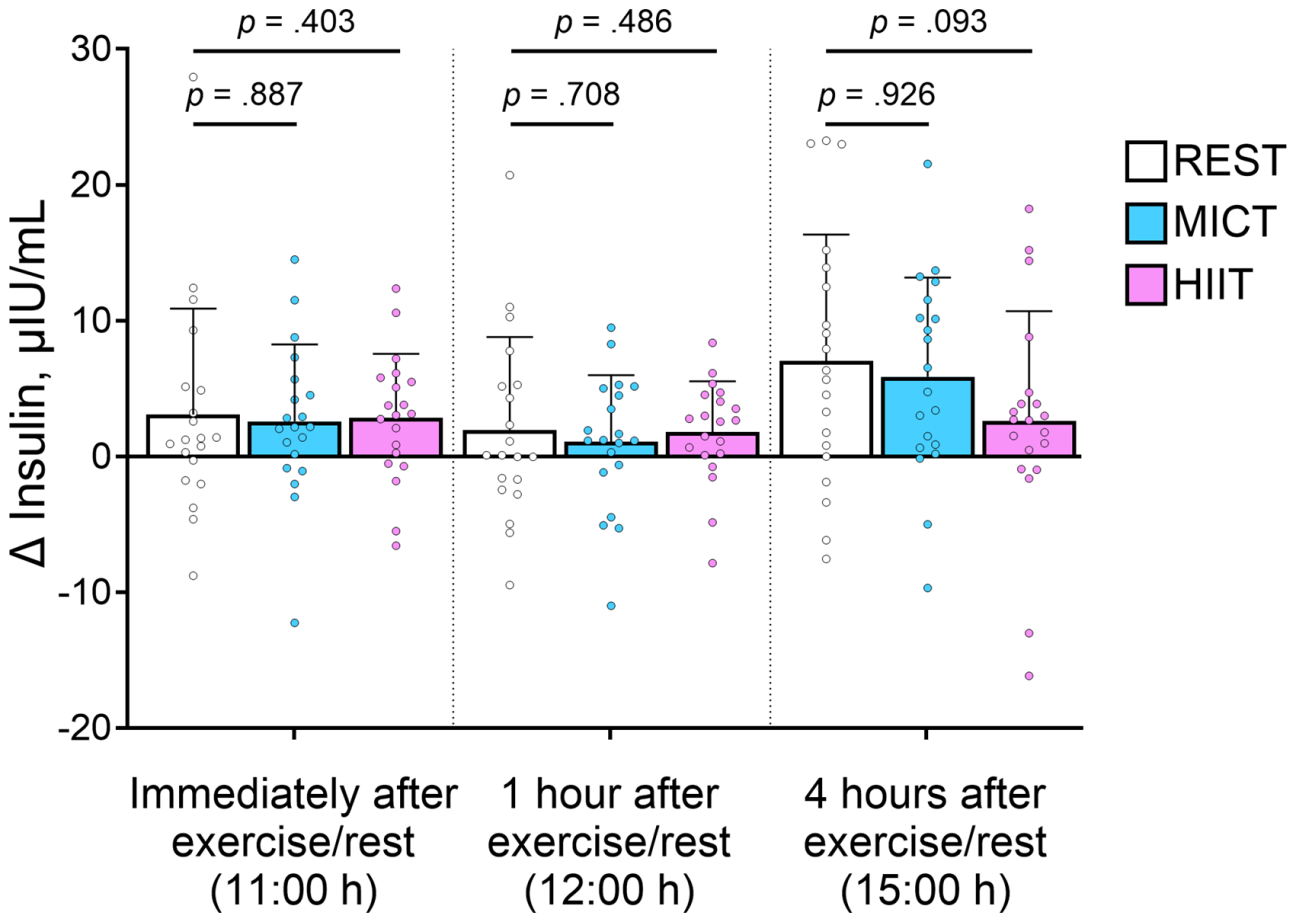
Change in insulin concentrations in each condition, compared with the sample obtained at 07:00 h. Bars show mean observed concentrations, error bars show standard deviations, and symbols show individual data. *p*-values represent the interaction between time and moderate-intensity continuous training (MICT)/high-intensity interval training (HIIT), compared with no exercise (REST), from linear mixed model, and therefore show the change from baseline.

There was no statistically significant effect of either exercise condition on human milk insulin concentration at any of the sampling time-points (Figure 3). However, there was a tendency (*p* = 0.093) of attenuated increase in insulin concentrations 4 h after exercise after HIIT, compared with the REST condition (Figure 3 and Table 3). Compared with the milk collected at 07:00 h, insulin concentrations were higher in the samples collected immediately after exercise (11:00 h) and in those collected 4 h after exercise (15:00 h, Table 3). The estimated effect of MICT and HIIT at each time-point on can be found by multiplying the exponentiated beta coefficients (estimate) for the main effect of time at each time-point with the exponentiated estimate for the interaction between time and exercise. The estimated relative change from the first to the last time-point was 72% (e^0.54^) for REST, 70% (e^0.54^* e^−0.01^) for MICT, and 41% (e^0.54^* e^−0.20^) for HIIT.

**Table 3:** Effect of time and interactions between time and conditions on log-transformed human milk insulin concentrations compared with a sample obtained at 07:00 h.

Main effect of time, log-transformed values			
	Estimate	95% confidence interval	*p*
11:00 h	0.26	0.07 to 0.45	0.009
12:00 h	0.18	−0.01 to 0.38	0.065
15:00 h	0.54	0.35 to 0.73	<0.001

Interaction between time and exercise, log-transformed values			
	Estimate	95% confidence interval	*p*
11:00 h × MICT	0.02	−0.22 to 0.25	0.887
12:00 h × MICT	−0.05	−0.28 to 0.19	0.708
15:00 h × MICT	−0.01	−0.25 to 0.23	0.926
11:00 h × HIIT	0.10	−0.14 to 0.34	0.403
12:00 h × HIIT	0.08	−0.15 to 0.34	0.486
15:00 h × HIIT	−0.20	−0.44 to 0.03	0.093

The interaction between time-points and moderate-intensity continuous training (MICT) and high-intensity interval training (HIIT) are compared with no exercise (REST). Data from 20 participants are estimated differences, corresponding 95% confidence interval (CI), and *p*-values from linear mixed model. The intercept was 2.1.

## 4 Discussion

Based on the well-established acute effect of exercise on circulating insulin levels (8), we hypothesized that insulin concentrations would also decline in human milk after exercise. However, we found no clear evidence of any acute effects of MICT or HIIT on insulin concentrations. Independent of test condition, insulin concentrations were higher 2 h after lunch compared with the fasted state. After HIIT, there was a tendency of attenuated increase in human milk insulin concentrations at 2 h after lunch, indicating that HIIT might reduce the postprandial increase in insulin.

Insulin is a peptide hormone secreted by the pancreas with pleiotropic effects on the body, including an essential role in glucose homeostasis and liver metabolism. Its presence in human milk has been known for 50 years (19). However, the impact of insulin in human milk on infant metabolism, appetite regulation, and weight gain is still not well understood. There are several mechanisms by which it is biologically plausible that insulin can be absorbed into the infant’s circulation (20). The highly permeable infant gastrointestinal tract expresses insulin receptors (21), which suggests that insulin may act locally or be absorbed into the circulation and mediate metabolism in other organs or tissues (20). As such, enteral insulin is reported to have beneficial local effects in the infant gut, promoting intestinal maturation (22).

Studies with data from humans on associations between human milk insulin and infant weight have shown conflicting results (10–14). In children from mothers with diabetes during pregnancy (type 1 or gestational), early ingestion of human milk from their biological mother resulted in increased prevalence of obesity at 2 years of age, compared with children who received banked milk from nondiabetic mothers (23). This finding may be attributed to several nutritional or other bio-active components that differ in the milk of mothers with and without diabetes, with insulin being one candidate. Human milk from mothers with diabetes contains several-fold increased concentrations of insulin (24), and human milk insulin concentrations are strongly correlated with circulating insulin concentrations also in mothers without diabetes (25). As exercise is a potent stimulus for increased insulin sensitivity, it is biologically plausible that human milk insulin concentrations will decrease after a bout of exercise.

Limitations to our study include the relatively small sample size and allowing the participants to breastfeed their infants on demand on the test days. Even if we only included 20 participants, the cross-over design of the study increases the statistical power of our analysis as each person serve as its own control. We did not control for an order effect of the conditions in our analysis, since we assumed that there would be no such effect. Due to logistical factors, there was some variance in the interval between the conditions. Most of the participants completed the three conditions 7 days apart, with 4 days being the shortest interval (for one participant) and 14 days the longest (for one participant). We believe the washout period between the conditions was sufficient to prevent any carryover effects and not long enough to induce a compositional change in human milk due to different lactation stages. The standardisation of dietary intake on the day prior to and on test days was based on self-reported intake. Even if the participants recorded what they consumed on the first condition and we asked them to repeat this at the subsequent conditions, we cannot rule out that some deviated from their reported intake.

Compared with the first milk sample obtained in the fasted state at 07:00 h, insulin concentrations were higher at later time-points. There was a tendency, albeit not statistically significant, for a dampening of the postprandial increase in human milk insulin concentrations 4 h after HIIT. Our study is the first investigation of the acute effects of exercise on human milk insulin concentrations, thus it is hard to compare our findings with previous research. However, a single session of HIIT has been shown to improve postprandial glucose tolerance (26), and the somewhat lower insulin concentrations in human milk after HIIT may be due to increased muscle glucose uptake in skeletal muscle in the hours after the session (27). The increase in insulin sensitivity following exercise reduces the need for circulating insulin to maintain glucose homeostasis after a meal. Together with our previous findings of increased human milk adiponectin concentrations 1 h after HIIT (6), the present findings signal a need for more in-depth research on the effect of exercise on human milk composition. Future studies should determine both the acute effects of a single exercise session and chronic effects induced by exercise training on human milk composition. Importantly, the potential impact of exercise-induced modifications to human milk for infant growth and metabolism must be investigated in longitudinal studies.

## Data Availability

The original contributions presented in the study are included in the article/Supplementary material, further inquiries can be directed to the corresponding author.

## References

[1] World Health Organization. (2024). Obesity and overweight 2024. Available at: https://www.who.int/news-room/fact-sheets/detail/obesity-and-overweight. (Accessed March 1, 2024)

[2] Voerman E Santos S Patro Golab B Amiano P Ballester F Barros H. Maternal body mass index, gestational weight gain, and the risk of overweight and obesity across childhood: an individual participant data meta-analysis. PLoS Med. (2019) 16:e1002744. doi: 10.1371/journal.pmed.1002744, PMID: 30742624 PMC6370184

[3] Mameli C Mazzantini S Zuccotti GV. Nutrition in the first 1000 days: the origin of childhood obesity. Int J Environ Res Public Health. (2016) 13:838. doi: 10.3390/ijerph13090838, PMID: 27563917 PMC5036671

[4] Gregg B Ellsworth L Pavela G Shah K Berger PK Isganaitis E. Bioactive compounds in mothers milk affecting offspring outcomes: a narrative review. Pediatr Obes. (2022) 17:e12892. doi: 10.1111/ijpo.12892, PMID: 35060344 PMC9177518

[5] Moholdt T Stanford KI. Exercised breastmilk: a kick-start to prevent childhood obesity? Trends Endocrinol Metab. (2023) 35:23–30. doi: 10.1016/j.tem.2023.08.019, PMID: 37735048 PMC11005327

[6] Holmen M Giskeødegård GF Moholdt T. High-intensity exercise increases breast milk adiponectin concentrations: a randomised cross-over study. Front Nutr. (2023) 10:1275508. doi: 10.3389/fnut.2023.1275508 38164413 PMC10757973

[7] Schneider-Worthington CR Bahorski JS Fields DA Gower BA Fernández JR Chandler-Laney PC. Associations among maternal adiposity, insulin, and adipokines in circulation and human milk. J Hum Lact. (2021) 37:714–22. doi: 10.1177/0890334420962711, PMID: 33035124 PMC8276526

[8] Richter EA Sylow L Hargreaves M. Interactions between insulin and exercise. Biochem J. (2021) 478:3827–46. doi: 10.1042/BCJ20210185, PMID: 34751700

[9] Hashemi Javaheri FS Karbin K Senobari MA Hakim HG Hashemi M. The association between maternal body mass index and breast milk composition: a systematic review. Nutr Rev. (2024) 83:83–111. doi: 10.1093/nutrit/nuad174, PMID: 38273741

[10] Christensen SH Lewis JI Larnkjær A Frøkiær H Allen LH Mølgaard C. Associations between maternal adiposity and appetite-regulating hormones in human milk are mediated through maternal circulating concentrations and might affect infant outcomes. Front Nutr. (2022) 9:1025439. doi: 10.3389/fnut.2022.1025439, PMID: 36407523 PMC9673480

[11] Sims CR Lipsmeyer ME Turner DE Andres A. Human milk composition differs by maternal BMI in the first 9 months postpartum. Am J Clin Nutr. (2020) 112:548–57. doi: 10.1093/ajcn/nqaa098, PMID: 32401302 PMC7458771

[12] Cheema AS Stinson LF Rea A Lai CT Payne MS Murray K. Human milk lactose, insulin, and glucose relative to infant body composition during exclusive breastfeeding. Nutrients. (2021) 13:3724. doi: 10.3390/nu13113724, PMID: 34835980 PMC8625960

[13] Nuss H Altazan A Zabaleta J Sothern M Redman L. Maternal pre-pregnancy weight status modifies the influence of PUFAs and inflammatory biomarkers in breastmilk on infant growth. PLoS One. (2019) 14:e0217085. doi: 10.1371/journal.pone.0217085, PMID: 31141526 PMC6541358

[14] Fields DA Demerath EW. Relationship of insulin, glucose, leptin, IL-6 and TNF-α in human breast milk with infant growth and body composition. Pediatr Obes. (2012) 7:304–12. doi: 10.1111/j.2047-6310.2012.00059.x, PMID: 22577092 PMC3393795

[15] Dewey KG Lovelady CA Nommsen-Rivers LA McCrory MA Lönnerdal B. A randomized study of the effects of aerobic exercise by lactating women on breast-milk volume and composition. N Engl J Med. (1994) 330:449–53. doi: 10.1056/NEJM199402173300701, PMID: 8289849

[16] Be'er M Mandel D Yelak A Gal DL Mangel L Lubetzky R. The effect of physical activity on human milk macronutrient content and its volume. Breastfeed Med. (2020) 15:357–61. doi: 10.1089/bfm.2019.0292, PMID: 32267727

[17] Berglund IJ Soras SE Relling BE Lundgren KM Kiel IA Moholdt T. The relationship between maximum heart rate in a cardiorespiratory fitness test and in a maximum heart rate test. J Sci Med Sport. (2019) 22:607–10. doi: 10.1016/j.jsams.2018.11.018, PMID: 30527685

[18] Craig CL Marshall AL Sjöström M Bauman AE Booth ML Ainsworth BE. International Physical Activity Questionnaire: 12-country reliability and validity. Med Sci Sports Exerc. (2003) 35:1381–95. doi: 10.1249/01.MSS.0000078924.61453.FB, PMID: 12900694

[19] Cevreska S Kovacev VP Stankovski M Kalamaras E. The presence of immunologically reactive insulin in milk of women, during the first week of lactation and its relation to changes in plasma insulin concentration. God Zb Med Fak Skopje. (1975) 21:35–41. 1213543

[20] Fields DA Schneider CR Pavela G. A narrative review of the associations between six bioactive components in breast milk and infant adiposity. Obesity. (2016) 24:1213–21. doi: 10.1002/oby.21519, PMID: 27151491 PMC5325144

[21] Ménard D Corriveau L Beaulieu JF. Insulin modulates cellular proliferation in developing human jejunum and colon. Biol Neonate. (1999) 75:143–51. doi: 10.1159/000014090, PMID: 9925901

[22] Mank E Sáenz de Pipaón M Lapillonne A Carnielli VP Senterre T Shamir R. Efficacy and safety of enteral recombinant human insulin in preterm infants: a randomized clinical trial. JAMA Pediatr. (2022) 176:452–60. doi: 10.1001/jamapediatrics.2022.0020, PMID: 35226099 PMC8886453

[23] Plagemann A Harder T Franke K Kohlhoff R. Long-term impact of neonatal breast-feeding on body weight and glucose tolerance in children of diabetic mothers. Diabetes Care. (2002) 25:16–22. doi: 10.2337/diacare.25.1.16, PMID: 11772895

[24] Jovanovic-Peterson L Fuhrmann K Hedden K Walker L Peterson CM. Maternal milk and plasma glucose and insulin levels: studies in normal and diabetic subjects. J Am Coll Nutr. (1989) 8:125–31. doi: 10.1080/07315724.1989.10720287, PMID: 2651503

[25] Young BE Patinkin Z Palmer C de la Houssaye B Barbour LA Hernandez T. Human milk insulin is related to maternal plasma insulin and BMI: but other components of human milk do not differ by BMI. Eur J Clin Nutr. (2017) 71:1094–100. doi: 10.1038/ejcn.2017.75, PMID: 28513622 PMC5587359

[26] Gillen JB Little JP Punthakee Z Tarnopolsky MA Riddell MC Gibala MJ. Acute high-intensity interval exercise reduces the postprandial glucose response and prevalence of hyperglycaemia in patients with type 2 diabetes. Diabetes Obes Metab. (2012) 14:575–7. doi: 10.1111/j.1463-1326.2012.01564.x, PMID: 22268455

[27] Mikines KJ Sonne B Farrell PA Tronier B Galbo H. Effect of physical exercise on sensitivity and responsiveness to insulin in humans. Am J Phys. (1988) 254:E248–59. doi: 10.1152/ajpendo.1988.254.3.E248, PMID: 3126668

