# Supplementary material for: Acute effect of endurance exercise on human milk insulin concentrations: a randomised cross-over study
Source: Front Nutr. 2025 Jan 27;11:1507156. doi: 10.3389/fnut.2024.1507156 (PMC11841457; doi:10.3389/fnut.2024.1507156)
Supplement: Supplementary file 1 [file Data_Sheet_1.PDF]

Supplementary Table 1. Insulin concentrations (in µIU/mL) for each participant and time-point

ID	REST_7h	REST_11h	REST_12h	REST_15h	MICT_7h	MICT_11h	MICT_12h	MICT_15h	HIIT_7h	HIIT_11h	HIIT_12H	HIIT_15h
1	16,587	17,931	14,883	24,501	40,857	39,992	39,689	31,168	27,672	22,172	22,805	30,322
2	10,127	8,098	8,519	33,111	20,803	8,538	9,795	34,501	20,641	14,057	12,781	7,631
3	6,497	4,731	4,051	4,602	4,275	4,426	5,269	7,672	4,444	4,691	4,652	8,305
4	5,778	8,974	11,054	20,969	7,029	9,072	11,515	20,270	12,107	19,299	16,814	27,308
5	13,893	9,263	8,282	7,732	8,625	7,552	3,326	10,125	8,354	7,825	7,587	6,725
6	17,320	17,629	14,532	13,944	6,796	11,312	11,804	7,673	5,510	10,603	11,638	8,224
7	14,882	11,092	9,911	23,943	6,091	11,758	14,363	10,849	9,729	20,304	18,096	27,978
8	4,832	6,063	4,931	28,083	4,684	6,844	6,606	17,539	6,920	6,196	7,005	5,930
9	8,388	9,305	8,359	11,674	9,542	12,453	11,207	16,076	6,865	12,985	5,336	11,560
10	5,061	5,820	5,039	6,823	5,634	6,668	6,797	15,758	6,685	9,442	12,019	9,679
11	4,713	7,306	4,812	5,505	3,495	4,883	3,807	12,117	4,218	10,015	6,872	3,282
12	7,635	12,759	12,786	13,977	9,813	13,986	10,964	9,674	3,537	6,668	6,095	7,399
13	6,123	17,684	13,885	20,021	5,787	17,286	15,276	27,328	7,468	11,216	10,468	16,251
14	4,257	3,957	6,577	8,797	3,185	6,009	4,389	6,198	6,555	8,648	7,217	8,066
15	8,686	21,102	19,701	14,344	9,316	18,097	8,688	9,505	9,059	21,421	13,592	9,538
16	8,087	9,480	9,200	8,070	5,952	8,129	9,431	6,595	4,863	10,351	7,628	6,639
17	19,231	10,443	9,748	11,686	11,091	8,108	6,603	6,078	7,941	6,124	9,060	11,211
18	3,367	8,243	7,656	13,047	13,474	11,452	8,389	22,764	4,745	7,780	6,247	19,156
19	4,391	32,326	25,114	27,434	11,620	26,130	16,900	21,808	53,191	56,997	57,217	37,043
20	6,244	15,531	16,515	18,724	7,379	14,679	12,523	18,905	10,169	11,014	13,681	11,129

REST = no activity, MICT = moderate-intensity continuous training, HIIT = high-intensity interval training
